# Live births after vaginal progesterone Cyclogest suppository versus Crinone gel for luteal phase support following cleavage and blastocyst cryopreserved embryo transfer (CET); a retrospective comparative study

**DOI:** 10.5935/1518-0557.20240076

**Published:** 2025

**Authors:** Dalia Alharbi, Marah Nadreen, Ayidah Albaiji, Dania Al-Jaroudi

**Affiliations:** 1 Reproductive Endocrine and Infertility Medicine Department. Women’s Specialized Hospital, King Fahad Medical City, Riyadh Second Health Cluster, Saudi Arabia; 2 Obstetrics and Gynecology Department. Women’s Specialized Hospital, King Fahad Medical City, Riyadh, Saudi Arabia

**Keywords:** luteal support, vaginal progesterone, Cyclogest, Crinone, IVF, frozen embryo transfer

## Abstract

**Objective:**

To compare the clinical outcomes, including pregnancy rate, live birth rate, and miscarriage rate between vaginal progesterone Cyclogest suppository and Crinone vaginal progesterone gel as LPS in frozen-thawed embryo transfer in Intra-Cytoplasmic Sperm Injection (ICSI) cycles.

**Methods:**

In this comparative retrospective chart review, 283 women who had frozen-thawed embryo transfer were assessed. The patients were divided into two groups based on the route of progesterone administration used as LPS. When the endometrial thickness reached ≥8mm, vaginal progesterone Cyclogest 400 mg/twice daily suppository was administered in one group; in another group, vaginal progesterone Crinone 8% 90 mg daily was administrated until a positive pregnancy test was confirmed. This was continued for 10-12 weeks after embryo transfer when fetal heart activity was detected by ultrasonography.

**Results:**

The patients’ characteristics in the two groups were matched and there was no significant difference. The biochemical and clinical pregnancy, miscarriage, and live birth rates were similar-4.7% *vs*. 2.7%, *p*=0.464; 26.1% *vs*. 23.3%, *p*=0.638; 13.3% *vs*. 9.6%, *p*=0.410; 15.6% *vs*. 16.4%, *p*=0.872, respectively; there was no statistically significant difference between the vaginal progesterone Cyclogest group and the Crinone progesterone group.

**Conclusions:**

Clinical pregnancy, biochemical pregnancy, miscarriage, and live birth rates were similar between both groups. Moreover, vaginal progesterone Cyclogest and Crinone 8% gel are equally effective in providing support during the luteal phase for both blastocysts and cleavage-stage embryos in CET.

## INTRODUCTION

Infertility is defined as “the failure to achieve a clinical pregnancy after 12 months or more of regular unprotected intercourse” (Zegers-Hochschild *et al.*, 2009). Embryos obtained through In Vitro Fertilization (IVF) or ICSI can be frozen and used in future cycles to prevent waste and increase the likelihood of pregnancy (Kalem *et al.*, 2016). Frozen Embryo Transfer (FET) was first reported in 1983, after which it became popular as an ART (Wang *et al.*, 2015). Progesterone is a crucial hormone that is necessary for the establishment and support of early pregnancy until the luteal-placental shift, which usually takes place at around 7-10 weeks of gestation (Csapo *et al.*, 1972; 1973a; 1973b). Progesterone for LPS is available in different routes, including vaginal, IMP, SC injection, oral, and rectal (Child *et al.*, 2018). Oral medications are frequently overlooked because they are not very effective and can cause unwanted side effects, such as drowsiness, dizziness, and headaches (Nahoul *et al.*, 1993; Simon *et al.*, 1993; Besins Healthcare, 2023). However, there are side effects for each route (Nahoul *et al.*, 1993; Simon *et al.*, 1993; Besins Healthcare, 2023).

During FET, the corpus luteum does not form naturally due to the absence of ovulation, which makes endometrial preparation for implantation of an embryo beginning from the follicular phase until the luteal phase an important step in ART (Nahoul *et al.*, 1993; Simon *et al.*, 1993; Child *et al.*, 2018; Besins Healthcare, 2023). LPS can be achieved directly by progesterone or by replacing the deficient Luteinizing Hormone (LH) with Gonadotrophin-Releasing Hormone (GnRH) agonists or Human Chorionic Gonadotropin (HCG) (Csapo *et al.*, 1973a). Factors such as the patient’s age, endometrial thickness, progesterone administration, embryo quality and its stage of development, as well as cryopreservation techniques can all impact pregnancy outcomes (Jiang *et al.*, 2019).

Much discussion and continuous research have been dedicated to finding the best way to administer progesterone. However, most of the work done has focused on stimulated IVF cycles. In addition, there is no single formulation or regimen that has been identified as superior (Nahoul *et al.*, 1993; Simon *et al.*, 1993; Lightman *et al.*, 1999; Haddad *et al.*, 2007; Glujovsky *et al.*, 2010; Kaser *et al.*, 2012; Shapiro *et al.*, 2014; van der Linden *et al.*, 2015; Besins Healthcare, 2023). In fact, a more recent meta-analysis concluded that there is insufficient evidence to recommend one progesterone regimen over another (Mackens *et al.*, 2017). The most convincing evidence thus far that compares IMP with vaginal progesterone for blastocyst stage CET comes from a study conducted by Devine *et al*. They found that the rates of ongoing pregnancy in cycles of blastocyst CET were significantly lower in those supported by vaginal Endometrin suppositories administered twice daily as compared to cycles supported by either IMP alone or a combination of IMP and Endometrin (Devine *et al.*, 2018). However, the generalizability of their study’s data was questionable, as it was uncertain whether the results obtained from using vaginal Endometrin twice daily and experiencing poorer clinical outcomes were applicable to all vaginal progesterone products and dosing schedules (Bakkensen *et al.*, 2020).

Further, Shiba *et al*. revealed that there were no statistically significant differences in clinical pregnancy, fetal heart, and miscarriage rates among all groups of vaginal progesterone Lutinus, Uterogestan, Luteum, and Crinone (Shiba *et al.*, 2020). Several studies (Schoolcraft *et al.*, 2000; Dal Prato *et al.*, 2008; Khan *et al.*, 2009; Zarutskie & Phillips, 2009; Kahraman *et al.*, 2010; Mitwally *et al.*, 2010; Yanushpolsky *et al.*, 2010; Silverberg *et al.*, 2012; van der Linden *et al.*, 2015) revealed that pregnancy and live birth outcomes have been found to be similar when using vaginal progesterone or IMP in fresh embryo transfer cycles. The various types of progesterone-including vaginal, intramuscular, subcutaneous, and rectal-are equally effective for supporting the luteal phase during IVF/ICSI (Wang *et al.*, 2021). However, it was advised that more research is required to determine the best LPS for FET cycles, while a few studies suggested that progesterone may increase the likelihood of a live birth (Wang *et al.*, 2021).

Vaginal progesterone is utilized in almost two-thirds of IVF cycles worldwide; in North America, 57% of cycles combine vaginal progesterone with IMP or use IMP alone. Vaginal progesterone is preferred by women due to the undesirable side effects of IMP (Baker *et al.*, 2014). Ng *et al.* (2003; 2007) conducted two studies to compare the side effects and patient convenience of two vaginal progesterone formulations used for LPS in stimulated IVF cycles. In the first study, there was no difference in perineal irritation observed between the use of Cyclogest suppositories and Crinone 8% gel (Ng *et al.*, 2003). In the second study, no difference was found in perineal irritation after the use of Cyclogest suppositories or Endometrin tablets for LPS, although more patients found administration of Endometrin tablets was difficult (Ng *et al.*, 2007). There is only one study thus far that compares different progesterone preparations, including Cyclogest (rectal route), Ultrogesten and Crinone (vaginal route) in stimulated IVF cycles. The same study reported similar plasma progesterone levels in all three groups despite the differences in the route of administration and no major side effects have been reported thus far (Tay & Lenton, 2005).

To the best of the researchers’ knowledge, only two studies have investigated progesterone administration in CET. One study compared the safety and efficacy of lowdose subcutaneous progesterone with vaginal progesterone for LPS in patients undergoing FET and found that biochemical and clinical pregnancy rates were higher in the vaginal progesterone group than those in the low-dose subcutaneous progesterone (Prolutex) group but were statistically unnoticeable (Aflatoonian & Mohammadi, 2021). The other study assessed the effectiveness of using IMP for LPS in cryopreserved blastocyst SET cycles. It was found that cycles in which Crinone 8% gel was used had a comparable chance of resulting in a live birth. Thus, the literature review revealed a knowledge gap in the difference of the outcomes of vaginal progesterone Cyclogest suppository *vs*. Crinone 8% gel as LPS in a CET in either blastocysts or cleavage stage embryos.

Therefore, the aim of this study is to compare the pregnancy rate, live birth rate, and miscarriage rate between vaginal progesterone Cyclogest suppository and vaginal progesterone Crinone 8% gel as LPS in frozen-thawed embryo transfer in ICSI cycles.

## MATERIALS AND METHODS

This single-center, retrospective comparative study was conducted at the King Fahad Medical City, Kingdom of Saudi Arabia, Riyadh. Data were obtained from the Reproductive Endocrine and Infertility Medicine Department (REIMD) at King Fahad Medical City, Riyadh Second Health Cluster from the Electronic Patient Information Chart (EPIC) and Health Information Management (HIM) electronic databases from January 2022 to December 2022. In this study, the medical records of 283 female patients who underwent CET cycles were retrieved and reviewed. The inclusion criteria in this study were women aged ≤ 42 years who have had primary and secondary infertility for ≥ 2 years and have previously underwent IVF with ICSI cycles in KFMC. Conventional IVF and PGS were excluded from the study.

Based on the LPS protocol, the patients were divided into two groups: the first group (n=211) received Cyclogest^®^ (LDCollins, UK) vaginal progesterone suppository and the second group (n=72) received Crinone^®^ (Merck Serono, Germany) vaginal progesterone gel. For the CET cycle, Follicle Stimulating Hormone (FSH), Luteinizing Hormone (LH), and Estradiol Hormone (E2) were measured on the second, third, or fourth menstrual cycle days. Ultrasound was also performed for all patients upon start. The starting dose ranged between 2mg/day and 6mg/day orally of Estradiol Valerat (Bayer Hispania, S. L., Spain) was used for Endometrial preparation. Patients came for a follow-up after 10-12 days to repeat the hormonal profile and ultrasound, when the maximum estimated endometrial thickness was measured on a sagittal plane.

A biochemical pregnancy was defined as a pregnancy with β-hCG >50IU/L 14 days after the embryo transfer. A clinical pregnancy was defined as a pregnancy with fetal heart activity on transvaginal ultrasonography six weeks after positive B-HCG. A miscarriage was defined as the loss of pregnancy before the twentieth week of gestation, while an ongoing pregnancy was considered as a pregnancy that continued beyond the twelfth week of gestation.

When the endometrial thickness was ≥ 8 mm, women in the first group received vaginal suppository progesterone (Cyclogest 400mg twice per day) and those in the second group received vaginal progesterone gel (Crinone 8%, 90mg daily). For cleavage-stage embryos, LPS medication was given for three days then FET was performed on the fourth day after luteal support. For the blastocyst stage, LPS medication was given for five days then FET was performed on the sixth day. LPS was continued until a positive pregnancy test was confirmed; thereafter, an ultrasound was performed to confirm clinical pregnancy. All embryo transfers were done under ultrasound guidance using a Wallace catheter (Cooper Surgical, Trumbull, CT, USA).

### Ethical considerations

This study was approved by the Institutional Review Board (IRB) of King Fahad Medical City, Kingdom of Riyadh, Saudi Arabia (IRB No: 00010471, 2023).

### Statistical analysis

For the categorical variables, the frequency analysis, which is expressed in percentage, was used. In contrast, continuous variables were reported in either the mean ± SD or median and interquartile range. Intergroup comparisons were determined by a t-test or Mann-Whitney U-test for continuous variables, as appropriate, and by utilizing the Chi-squared test or Fisher exact test for categorical variables. All the statistical inferences were drawn at a 95% confidence interval. SPSS version 22.0 (IBM, Armonk, NY, USA) software was used for data analysis.

## RESULTS

This study included 283 women between the ages of 23 and 42 years, among which 211 (74.3%) were in the group who received the Cyclogest vaginal progesterone suppository and 72 (25.7%) were in the group that received the Crinone vaginal progesterone gel. Regarding the patients’ characteristics, no statistically significant difference was observed between the two groups for almost all the characteristics, except for Endometrial thickness (Table 1). The age (year) of the Cyclogest group was 33.9 ± 4.6 and that of the Crinone group was 34.1±5.1; the difference between the two groups was not significant (*p*=0.845). The BMI (kg/m^2^) of the Cyclogest group was 28.4±5.1 and that of the Crinone group was 27.3±5.2; the difference between the two was also not significant (*p*=0.119). Basal FSH level in the Cyclogest group was between 3.0 and 18.9IU/L, with a mean level of 5.8±1.9IU/L, while that of the Crinone group was between 2.4 and 15.7IU/L, with a mean level of 5.9±2.3IU/L; the difference was not significant (*p*=0.643). Basal LH level in the Cyclogest group was between 0.5 and 22.6IU/L, with a mean level of 5.3±3.1IU/L, while that of the Crinone group was between 1.1 and 15.8IU/L, with a mean level of 5.4±2.8IU/L; however, the difference was not significant (*p*=0.866).

**Table 1:** Embryo and CET cycle characteristics.

Patients Characteristic	Description	Cyclogest group N (%) = 211 (74.3%)	Crinone group N (%) = 72 (25.7%)	p-value
**Medication**	**Estradiol: N (Mean)**	205 (97.2)	72 (98.6)	0.682
		
	**Other: N (Mean)**	6 (2.8)	1 (1.4)	
**Endometrial preparation medication duration**	**min-max**	9-21	9-21	0.114
	**Mean ± SD**	14.9±2.0	15.3±2.2	
	**Median (P25-P75)**	15 (14-16)	15 (14-17)	
**Embryo type**	**Cleavage stage embryo: N (Mean)**	139 (65.9)	40 (54.8)	0.091
		
	**Blastocyst embryo:** **N (Mean)**	72 (34.1)	33 (45.2)	
**Number of transferred embryos**	**1: N (Mean)**	47 (22.3)	17 (23.3)	0.933
	**2: N (Mean)**	150 (71.1)	52 (71.2)	
	**3: N (Mean)**	14 (6.6)	4 (5.5)	
**Number of frozen embryos**	**Min-max** **Mean ± SD** **Median (P25-P75)**	1 - 16 4±3 3 (2-6)	1-12 3±3 2 (2-4)	0.118
**Intervention duration**	**min-max**	2-8	2-7	0.587
	**Mean ± SD**	4.6±1.1	4.7±1.2	
	**Median (P25 - P75)**	5 (4 - 6)	5 (4 - 5)	

With regard to the embryo and CET cycle characteristics (Table 2), the most frequently used medication for endometrial priming in both the Cyclogest group (205, 97.2%) and Crinone group (72, 98.6%) was Estradiol Valerate; no significant difference was found between the groups (*p*=0.682). Only seven patients had recombinant FSH for priming. The endometrial preparation medication duration in either of the study groups ranged between 9 and 21 days; the mean duration period in the Cyclogest and Crinone groups was 14.9±2.0 and 15.3±2.2, respectively, and the difference was not significant (*p*=0.114).

**Table 2:** Comparison of pregnancy outcomes between the two groups.

Variable	Category	Cyclogest group N (%) = 211 (74.3%)	Crinone group N (%) = 72 (25.7%)	p-value
**Biochemical pregnancy**	**Yes**	10 (4.7)	2 (2.7)	0.464
	**No**	201 (95.3)	71 (97.3)	
**Clinical pregnancy**	**Yes**	55 (26.1)	17 (23.3)	0.638
	**No**	156 (73.9)	56 (76.7)	
**Miscarriage**	**Yes**	28 (13.3)	7 (9.6)	0.410
	**No**	183 (86.7)	66 (90.4)	
**Live birth**	**Yes** **No**	33 (15.6) 178 (84.4)	12 (16.4) 61 (83.6)	0.872

In the Cyclogest group, 139 patients (65.9%) had cleavage-stage embryo transfer and 72 (34.1%) had blastocyst embryo transfer. In contrast, in the Crinone group, 40 of the patients (54.8%) had cleavage-stage embryo transfer and 33 (45.2%) had blastocyst embryo transfer.

For both embryo types, the median value was higher for the Cyclogest group, however, the difference was not significant (*p*=0.091). Moreover, 1-3 transferred embryos were placed in each group, and the highest median value was present when two embryos were placed in the two groups-it was 150 (71.1%) for the Cyclogest group and 52 (71.2%) for the Crinone group. Each group had similar distributions and the difference was not significant (*p*=0.933). The minimum and maximum number of frozen embryos in the Cyclogest group were 1 and 16, with a median value of 3 (2-6) embryos. In contrast, the minimum and maximum in the Crinone group were 1 and 12, respectively, with a median value of 2 (2-4) embryos; the difference was not significant (*p*=0.118). The intervention duration in the Cyclogest group ranged between two and eight days, with a mean duration of 4.6±1.1 days. In contrast, it ranged between two and seven days in the Crinone group, with a mean duration of 4.7±1.2 days; the difference between the two groups was not significant (*p*=0.587). However, a significant difference was observed in the endometrial thickness (mm) where it was between 4 mm and 19 mm in the Cyclogest group, with a median value of 8 mm, and between 4 mm and 13 mm for the Crinone group, with a median value of 8 mm (*p*=0.046).

**Figure 1:**
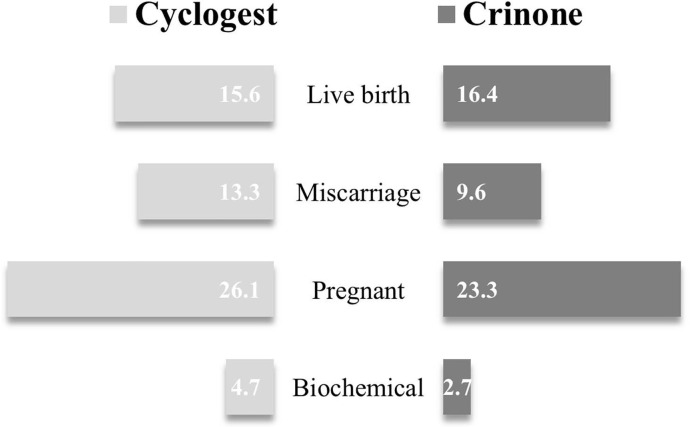
The variations in the present clinical outcomes of the Cyclogest and Crinone groups.

The clinical results revealed that biochemical and clinical pregnancy rates were higher in the Cyclogest group than those in the Crinone group, but the difference was statistically insignificant (Table 3 and Figure 1). Further, Biochemical pregnancy was seen among 10 (4.7%) subjects in the Cyclogest group and 2 (2.7%) subjects in the Crinone group; the difference between the two groups was not significant (*p*=0.464). The total number of clinical pregnancy cases in the Cyclogest group was 55 (26.1%), while the total number of clinical pregnancy cases in the Crinone group was 17 (23.3%) (*p*=0.638). However, the number of miscarriages in the Cyclogest group were 28 (13.3%), which exceeded the number of miscarriages in the Crinone group at 7 (9.6%); however, the difference between the two groups was not significant (*p*=0.410). Furthermore, the number of live births in the Cyclogest group were 33 (15.6%) and those in the Crinone group were 12 (16.4%); the difference between the two groups was not significant (*p*=0.872).

**Table 3:** Comparison of patient characteristics between the two groups.

Patients’ Characteristics	Description	Cyclogest group N (%) = 211 (74.3%)	Crinone group N (%) = 72 (25.7%)	p-value
**Age (year)**	**min – max**	25–42	23–42	0.846
	**Mean ± SD**	33.9±4.6	34.1±5.1	
	**Median (P25–P75)**	34 (30–38)	35 (30–38)	
**BMI (kg/m^2^)**	**min–max**	15.69–41.5	16.06 - 37.81	0.119
	**Mean ± SD**	28.4±5.1	27.3±5.2	
	**Median (P25–P75)**	28 (24.88–32.44)	27.64 (24.03–31.09)	
**Endometrial thickness** **(mm)**	**min–max**	4–19	4–13	0.046
	**Mean ± SD**	8.8±1.5	8.4±1.6	
	**Median (P25–P75)**	8 (8–10)	8 (8–9)	
**Basal FSH (IU/L)**	**min–max**	3.0–18.9	2.4–15.7	0.643
	**Mean ± SD**	5.8±1.9	5.9±2.3	
	**Median (P25–P75)**	5.3 (4.7–6.6)	5.1 (4.6–6.6)	
**Basal LH (IU/L)**	**min–max**	0.5–22.6	1.1–15.8	0.866
	**Mean ± SD**	5.3±3.1	5.4±2.8	
	**Median (P25–P75)**	4.6 (3.3–6.7)	4.9 (3.3–6.5)	

## DISCUSSION

The present study compared the clinical outcomes- including pregnancy rate, live birth rate, and miscarriage rate-between two groups of women that received Cyclogest vaginal progesterone suppository and Crinone vaginal progesterone gel as LPS in CET cycles for both cleavage and blastocyst stage. Our data analysis revealed higher median values for the Cyclogest group for all the clinical outcomes than those for the Crinone group. However, the differences between the two vaginal progesterone routes were not statistically different. Hence, our study finding is in line with most of the literature on stimulated IVF cycles as well as the two founded studies that directed toward the use of frozen-thawed embryo transfer (Bakkensen *et al.*, 2020; Aflatoonian & Mohammadi, 2021). A statistical difference in endometrial thickness between the two groups with Cyclogest vaginal progesterone and Crinone vaginal progesterone gel was found. This difference can be attributed to factors other than vaginal progesterone treatments, as patients were typically given these treatments when their endometrial thickness was already appropriate.

According to Wang *et al.*, the various types of progesterone-including vaginal, IMP, SC, and rectal-are equally effective for supporting the luteal phase during IVF/ICSI (Wang *et al.*, 2021). Nevertheless, additional research is advised to ascertain the most effective means of supporting the luteal phase during FET cycles, as certain studies suggest that the use of progesterone may enhance the likelihood of achieving a successful live birth (Wang *et al.*, 2021). As mentioned previously, of the three studies that utilized the Cyclogest group as an intervention, one compared the convenience and side effects experienced by patients who used Cyclogest versus Crinone (Ng *et al.*, 2003; 2007) and the other experienced by patients who used Endometrin (Tay & Lenton, 2005). In the first study, 60 infertile patients were randomly assigned to receive either Cyclogest vaginal suppositories 400 mg twice daily or Crinone 8% vaginal gel once daily for 14 days as luteal support and no difference was found in perineal irritation after Cyclogest suppositories or Crinone 8% gel (Ng *et al.*, 2003). In the second study, 132 infertile patients were randomly assigned to receive either Cyclogest 400 mg or Endometrin 100 mg twice daily for 14 days on the day of embryo transfer. Although there was a trend of fewer patients with perineal irritation in the Endometrin group, no significant differences in perineal irritation were found on days 6 and 16 after embryo transfer between the two groups (Ng *et al.*, 2007). In the third study, three different progesterone preparations were administered to participants. These were Cyclogest 400 mg (n=35) administered rectally, Ultrogesten 200 mg, 400 mg, or 600 mg (n=55), and Crinone (n=36) administered vaginally (Tay & Lenton, 2005). The study reported similar plasma progesterone levels among all three groups, ranging between 23 nmol/L -26 nmol/L. However, Polyzos *et al.* (2010) acknowledged that a potential source of clinical heterogeneity might be present in the studies of Tay & Lenton (2005) and (Ng *et al.*, 2003) due to the fact that the day of initiation of LPS was not consistent between the trials (Polyzos *et al.*, 2010). The common practices imply that LPS is continued until 10-12 weeks of gestation, as reported by Di Guardo *et al.* (2020) for more than half of the clinicians surveyed in their study, and this is the case in this study as we administered LPS to pregnant women until 12 weeks of gestation.

Two studies were directed to CET. Bakkensen *et al.* (2020) study included 1710 cycles, of which 1594 utilized IMP and 116 utilized 8% Crinone gel. The demographic and cycle characteristics were similar between the two groups (Bakkensen *et al.*, 2020). However, there were no significant differences that resulted in similar rates of live birth (RR 0.91; 95% CI 0.73-1.13), biochemical pregnancy (RR 1.12, 95% CI 0.65-1.92), spontaneous miscarriage (RR 1.41, 95% CI 0.90-2.20), and clinical pregnancy (RR 1.00, 95% CI 0.86-1.17) (Bakkensen *et al.*, 2020). The other study by Aflatoonian and Mohammadi revealed that the subcutaneous aqueous progesterone (Prolutex) 25 mg daily has an equal effect as that of vaginal progesterone. The clinical and ongoing pregnancy rates were 22.2% with Prolutex and 28% with vaginal progesterone (*p*=0.581); no significant difference was reported in any of the secondary outcomes, including the implantation rate and miscarriage (Aflatoonian & Mohammadi, 2021).

Considering the findings presented in this discussion, it was evident that the effectiveness of the various routes of progesterone LPS has been studied extensively in the literature; however, no single route appears to be preferred during CET. In view of this, we argue that the patient’s preference should be considered-in addition to other social, legislative, and regulatory factors-to decide which route must be selected.

This study has several noteworthy strengths. It was conducted at a single center, where clinical and lab protocols remained consistent throughout the research period, which improved the generalizability of the findings. Additionally, the study is one of the few that focused on the CET cycle and is the only study, to the best of our knowledge, that compared the clinical outcome of vaginal progesterone Cyclogest versus Crinone in both cleavage and blastocyst embryo stages.

However, our study is limited by its retrospective design, and patients’ satisfaction data were not included nor collected from the patients subsequently by the authors. The reason behind that is if this data was collected later, it might have been influenced by the elapsed time between taking the medication and the data recording, during which the patients may have developed negative perceptions regarding the drug. Moreover, the number of Crinone cycles included in the study was relatively small compared to the Cyclogest cycles. Thus, there is a possibility that with a larger sample size, a few of the differences in the outcomes data may attain statistical significance. To further investigate these findings, a larger prospective study is required in which satisfaction data is recorded directly after the treatments.

## CONCLUSION

Clinical pregnancy, biochemical pregnancy, miscarriage, and live birth rates were similar between both groups. Moreover, vaginal progesterone Cyclogest and Crinone 8% gel are equally effective in providing support during the luteal phase for both blastocysts and cleavage-stage embryos in CET. These findings advance the understanding that there are no significant differences among various progesterone formulations when it comes to supporting implantation and pregnancy.
